# A Novel Psychovisual Threshold on Large DCT for Image Compression

**DOI:** 10.1155/2015/821497

**Published:** 2015-03-22

**Authors:** Nur Azman Abu, Ferda Ernawan

**Affiliations:** ^1^Faculty of Information and Communication Technology, Universiti Teknikal Malaysia Melaka, 76100 Melaka, Malaysia; ^2^Faculty of Computer Science, Universitas Dian Nuswantoro, Semarang 50131, Indonesia

## Abstract

A psychovisual experiment prescribes the quantization values in image compression. The quantization process is used as a threshold of the human visual system tolerance to reduce the amount of encoded transform coefficients. It is very challenging to generate an optimal quantization value based on the contribution of the transform coefficient at each frequency order. The psychovisual threshold represents the sensitivity of the human visual perception at each frequency order to the image reconstruction. An ideal contribution of the transform at each frequency order will be the primitive of the psychovisual threshold in image compression. This research study proposes a psychovisual threshold on the large discrete cosine transform (DCT) image block which will be used to automatically generate the much needed quantization tables. The proposed psychovisual threshold will be used to prescribe the quantization values at each frequency order. The psychovisual threshold on the large image block provides significant improvement in the quality of output images. The experimental results on large quantization tables from psychovisual threshold produce largely free artifacts in the visual output image. Besides, the experimental results show that the concept of psychovisual threshold produces better quality image at the higher compression rate than JPEG image compression.

## 1. Introduction

Most digital cameras implement a popular block transform in image coding [1]. The sequential block-based coding is a popular technique since it is compact and easy to implement. In standard JPEG image compression, an image is compressed by one block of 8 × 8 pixels at a time. The block-based DCT coding has prevailed at reducing interpixel statistical redundancy [2]. However, in order to achieve high compression ratio, 8 × 8 image block size with default JPEG quantization tables produces discontinuity of intensities among adjacent image blocks. These discontinuities of the intensity image between two adjacent image blocks cause a visual artifact due to interblock correlations [3] in image reconstruction. Block transform coding always results in blocking artifact at low bit rate. Blocking artifact is one of the most annoying problems [4].

The blocking effect becomes visible within smooth regions where adjacent block is highly correlated with an input image. Since the 8 × 8 blocks of image pixels are coded separately, the correlation among spatially adjacent blocks provides boundary blocks when the image is reconstructed [5]. The artifact images in the compressed output are introduced by two factors. First, the transform coefficients coming out of the quantization process are rounded and then inadequately dequantized. Second, the blocking artifacts appear by the pixel intensity value discontinuities which occur along block boundaries [6]. These blocking artifacts are often visually observable.

This research pays a serious attention to the role of block size in transform image coding. Referring to JPEG image compression, the block of 8 × 8 image pixels based line coding has provided a low computational complexity. Previously, a compression scheme on 16 × 16 block has been investigated by Pennebaker and Mitchell [7] for image compression. This scheme does not provide an improvement on the image compression due to lack of progress on the central processing unit in terms of its computing power at the time. The larger 16 × 16 block requires an extra image buffering and a higher precision in internal calculations. Nowadays, the technology of the central processing unit grows rapidly in terms of its computing power. Therefore, two-dimensional image transform on larger blocks is now practically efficient to operate on image compression.

In the previous research, the psychovisual threshold has been investigated on 8 × 8 image block size [8–15]. This paper proposes psychovisual threshold on the large image block of 256 × 256 DCT in order to reduce significant blocking effect within the boundary of small image block. This paper also discusses the process and apparatus to generate 256 × 256 quantization tables via a psychovisual threshold.

The organization of this paper is as follows. The next section provides a brief overview on a psychoacoustic model.  Section 3 discusses a brief description of the 256 × 256 discrete cosine transform.  Section 4 explains the development of psychovisual threshold on the large discrete cosine transform in image compression.  Section 5 discusses a quality measurement on compressed output images.  Section 6 shows the experimental results of 256 × 256 quantization tables from psychovisual threshold in image compression. Lastly, Section 7 concludes this paper.

## 2. The Principle of Psychoacoustic Model 

**Figure 1:**
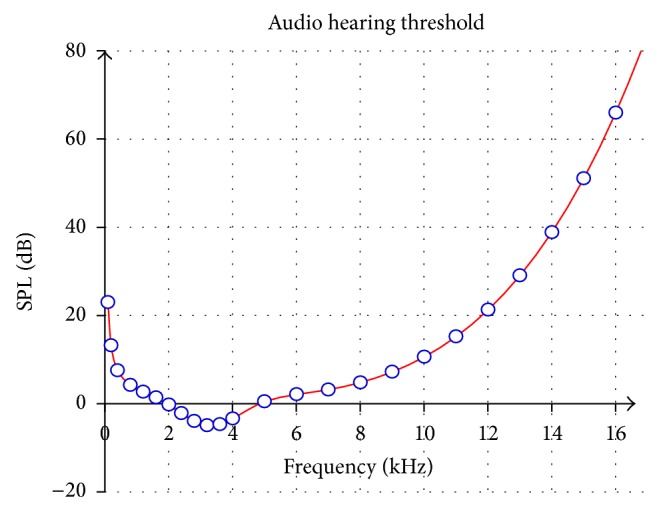
The absolute threshold of hearing under quiet condition.

Psychoacoustics is the study on how humans perceive sound or human hearing. Psychoacoustic studies show that the sound can only be heard at certain or higher sound pressure levels (SPL) across frequency order [16]. The psychoacoustics indicates that human hearing sensation has a selective sensitivity to different frequencies [17]. In the noise-free environment, the human ear audibility requires different loudness across various frequency orders. The sound loudness that the human audibility can hear is called the absolute hearing threshold [18] as depicted in Figure 1.

The principle of the psychoacoustic model by incrementing the sound pressure level one bark at a time has been used to detect audibility of human hearing threshold. The same principle of the psychoacoustic technique has been used to measure the psychovisual threshold in image compression by incrementing image frequency signal one-unit scale at a time. A block of image signals as represented by a transform coefficient at a given frequency is incremented one at a time on each frequency order. A psychovisual threshold has been conducted for image compression by calculating the just noticeable difference (JND) of the compressed image from the original image. This research will investigate the contribution of the transformed coefficient on each frequency order to the compressed output image. The average reconstruction error from the contribution of the DCT coefficients in image reconstruction will be the primitive of psychovisual threshold in image compression. This quantitative experiment has been conducted on 256 × 256 image blocks.

## 3. Discrete Cosine Transform 

**Figure 2:**
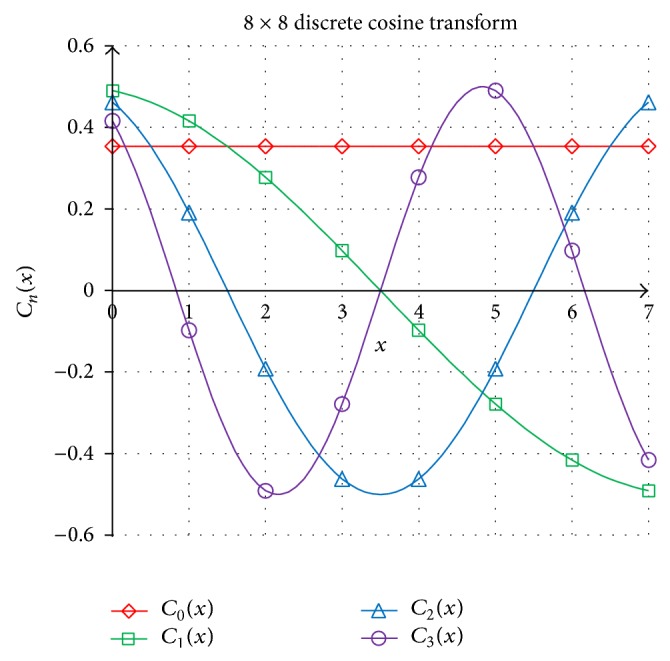
The first four 8 × 8 DCT of set *C*_*n*_(*x*) for *n* = 0, 1, 2, 3.

The two-dimensional discrete cosine transform [19] has been widely used in image processing applications. The DCT is used to transform the pixel values to the spatial frequencies. These spatial frequencies represent the detailed level of image information. The standard JPEG compression uses 8 × 8 DCT as shown in Figure 2 in image compression.

**Figure 3:**
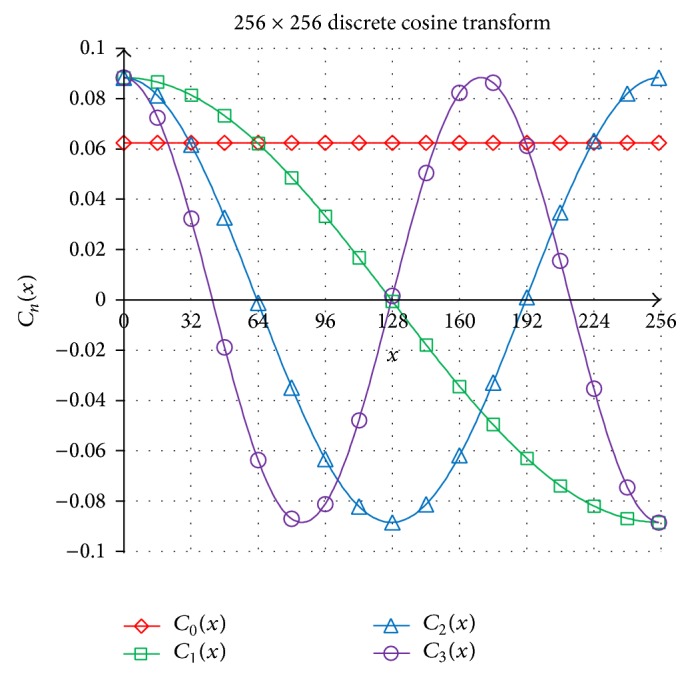
The first four 256 × 256 DCT of set *C*_*n*_(*x*) for *n* = 0, 1, 2, 3.

This paper proposes a large image block of *N* × *N* DCT set *C*_*n*_(*x*) of size *N* = 256 which can be generated iteratively as follows:

(1) C0(x)=1N,C1x=2Ncos⁡2x+11π2N,C2x=2Ncos⁡2x+12π2N,C3x=2Ncos⁡2x+13π2N,

for *x* = 0, 1, 2, …, *N* − 1. The first four one-dimensional 256 × 256 DCT above are depicted in Figure 3 for visual purposes.

The kernel for the DCT is derived from the following definition [20]:

(2) $$\begin{array}{c} g=\lambda \left(u\right)\cos\frac{\left(2x+1\right)u\pi }{2N}, \end{array}$$

where

(3) $$\begin{array}{c} \lambda \left(u\right)=\left\{\begin{array}{cc} \frac{1}{\sqrt{N}}, & u=0\\ \sqrt{\frac{2}{N}}, & u>0, \end{array}\right. \end{array}$$

for *x* = 0, 1, 2, …, *N* − 1 and *u* = 0, 1, 2, …, *N* − 1. The definition of the two-dimensional DCT of an input image *A* and output image *B* is given as follows [19]:

(4) $$\begin{array}{c} B_{pq}=\alpha_{p}\beta_{q}\overset{M-1}{\underset{m=0}{\sum }}\;‍\overset{N-1}{\underset{n=0}{\sum }}A_{mn}\cos\frac{\pi \left(2m+1\right)p}{2M}\cos\frac{\pi \left(2n+1\right)q}{2N}, \end{array}$$

for *p* = 0, 1, 2, …, *M* − 1 and *q* = 0, 1, 2, …, *N* − 1 where

(5) $$\begin{array}{c} \alpha_{p}=\left\{\begin{array}{cc} \frac{1}{\sqrt{M}}, & p=0\\ \sqrt{\frac{2}{M}}, & p>0 \end{array}\right.,\;\;\beta_{q}=\left\{\begin{array}{cc} \frac{1}{\sqrt{N}}, & q=0\\ \sqrt{\frac{2}{N}}, & q>0. \end{array}\right. \end{array}$$

The inverse of two-dimensional DCT is given as follows:

(6) $$\begin{array}{c} A_{pq}=\overset{M-1}{\underset{m=0}{\sum }}\;‍\overset{N-1}{\underset{n=0}{\sum }}\alpha_{p}\beta_{q}B_{mn}\cos\frac{\pi \left(2m+1\right)p}{2M}\cos\frac{\pi \left(2n+1\right)q}{2N}, \end{array}$$

for *p* = 0, 1, 2, …, *M* − 1 and *q* = 0, 1, 2, …, *N* − 1. The image input is subdivided into *M* × *N* blocks of image pixels. The DCT is used to transform each pixel in the 256 × 256 image block pixel into the frequency transform domain. The outputs of transforming 256 × 256 image blocks of frequency signals are 65536 DCT coefficients. The first coefficient in the upper left corner of the array basis function is called the direct current (DC) coefficient and the rest of the coefficients are called the alternating current (AC) coefficients. DC coefficient provides an average value over the 256 × 256 block domain.

## 4. Psychovisual Threshold on Large Discrete Cosine Transform 

**Figure 4:**
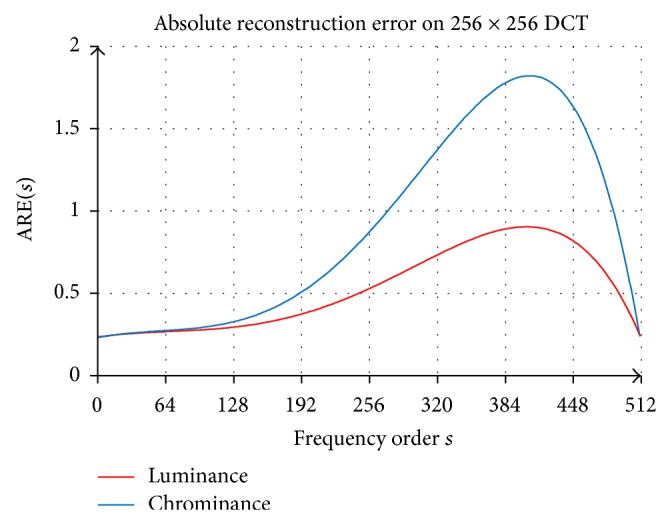
Average absolute reconstruction error of incrementing DCT coefficients on 256 × 256 DCT luminance and chrominance for 40 real images.

In this quantitative experiment, the DCT coefficients on each frequency order are incremented concurrently one at a time from 1 to 255. The impact of incrementing DCT coefficients one at a time is measured by average absolute reconstruction error (ARE). The contribution of DCT coefficients to the quality image reconstruction and compression rate is analyzed on each frequency order. In order to develop psychovisual threshold on 256 × 256 DCT, ARE on each frequency order is set as a smooth curve reconstruction error. An ideal average reconstruction error score of an incrementing DCT coefficient on each frequency order on luminance and chrominance for 40 real images is shown in Figure 4.

An ideal finer curve of ARE on a given order from zero to the maximum frequency order 510 for luminance and chrominance channels is presented by the red curve and blue curve, respectively. These finer curves of absolute reconstruction error are set as the psychovisual threshold on 256 × 256 DCT. The contribution of an ideal error reconstruction for each frequency order is determined by two factors, its contribution to the quality on image reconstruction and the bit rates on image compression. The smooth curve of ARE is interpolated by a polynomial that represents the psychovisual error threshold on 256 × 256 DCT in image compression. With reference to Figure 4, this paper proposes the new psychovisual thresholds on 256 × 256 DCT basis function for luminance *f*_*VL*_ and chrominance *f*_*VR*_ which are simplified as follows:

(7) fVLx=−0.0000000000001435x5+0.000000000011x4+0.000000046x3−0.000009x2+0.00088x+0.2352,fVRx=−0.000000000000457x5+0.000000000156x4+0.00000006x3−0.0000128x2+0.0012x+0.2309,

for *x* = 0, 1, 2,…, 510, where *x* is a frequency order on 256 × 256 image block. Further, these thresholds are used to generate smoother 256 × 256 quantization values for image compression. The 256 × 256 quantization table has 511 frequency orders from order 0 until order 510. The order 0 resides in the top left most corner of the quantization matrix index of *Q*(0,0). The first order represents the quantization value of *Q*(1,0) and *Q*(0,1). The second order represents *Q*(2,0), *Q*(1,1), and *Q*(0,2). For each frequency order, the same quantization value is assigned to them.

**Figure 5:**
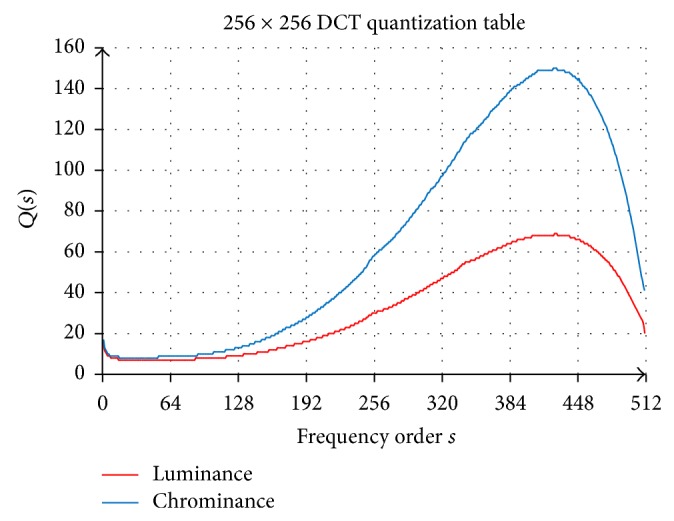
The 256 × 256 DCT quantization table for luminance and chrominance for image compression.

Due to the large size of these new 256 × 256 quantization values, it is not possible to present the whole quantization matrix within a limited space in this paper. Therefore, the 256 × 256 quantization values are presented by traversing the quantization table on each frequency order in zigzag pattern as shown in Table 1. Table 1 presents one-dimensional index of quantization table on each frequency order. Each index represents the quantization value at those frequency orders. The new finer quantization tables from psychovisual threshold on 256 × 256 DCT for luminance and chrominance are shown in Tables 2 and 3, respectively. The visualization of the whole quantization values on each frequency order from the psychovisual threshold for luminance and chrominance channels is depicted in Figure 5.

These new finer 256 × 256 quantization tables have been generated from the psychovisual threshold functions in (7). Each traversing array in zigzag pattern represents the quantization value on each quantization order from order 0 to order 510. This new smoother 256 × 256 quantization table for luminance is designed to take smaller value than a quantization table for chrominance. Any slight changes on respective frequency order in the luminance channel will generate significantly greater reconstruction error than a change in chrominance channel. The slight changes by the image intensity on the luminance channel will provide visible textures that can be perceived by human visual systems.

The contribution of the frequency signals to the reconstruction error is mainly concentrated in the low frequency order. Referring to Figure 1, the SPL values on frequency from 2 kHz to 5 kHz are significantly lower. These quantization tables follow the same pattern in order to capture the concept of the psychoacoustic absolute hearing threshold. The quantization values from the psychovisual threshold on chrominance channel are designed to be larger than the quantization values on luminance channel. The human visual system is less sensitive to the chrominance channels as they provide significantly irrelevant image information. The smoother quantization tables will be tested and verified in image compression.

## 5. Quality Measurement 

Two statistical evaluations have been conducted in this research project to verify the performances of psychovisual threshold in image compression. In order to gain significantly better performance, the image compression algorithm needs to achieve a trade-off between average bit rates and quality image reconstruction. The conventional quality assessments are employed in this paper. They are average absolute reconstruction error (ARE), means square error (MSE), and peak signal to noise ratio (PSNR). The average reconstruction error can be defined as follows:

(8) $$\begin{array}{c} \text{ARE}\left(s\right)=\frac{1}{\text{MNR}}\overset{M-1}{\underset{i=0}{\sum }}\;\overset{N-1}{\underset{j=0}{\sum }}\;\overset{R-1}{\underset{k=0}{\sum }}\left|g\left(i,j,k\right)-f\left(i,j,k\right)\right|, \end{array}$$

where the original image size *M* × *N* × *R* refers to the three RGB colors. The MSE calculates the average of the square of the error defined as follows [21]:

(9) $$\begin{array}{c} \text{MSE}=\overset{M-1}{\underset{i=0}{\sum }}\;\overset{N-1}{\underset{j=0}{\sum }}\;\overset{R-1}{\underset{k=0}{\sum }}{\left\|g(i,j,k)-f(i,j,k)\right\|}^{2}. \end{array}$$

The standard PSNR is used and calculated to obtain the measure of the quality of the image reconstruction. A higher PSNR means that the image reconstruction is more similar to the original image [22]. The PSNR is defined as follows:

(10) $$\begin{array}{c} \text{PSNR}=20\text{lo}{\text{g}}_{10}\left(\frac{{\mathrm{Max}}_{i}}{\sqrt{\text{MSE}}}\right)=10\text{lo}{\text{g}}_{10}\left(\frac{255^{2}}{\text{MSE}}\right), \end{array}$$

where *Max*⁡_*i*_ is the maximum possible pixel value of the image. Structural similarity index (SSIM), another measurement of image quality, is a method to measure quality by capturing the similarity between original image and compressed image [23]. The SSIM is defined as follows:

(11) $$\begin{array}{c} \text{SSIM}\left(x,y\right)={\left[l\left(x,y\right)\right]}^{\alpha }\cdot {\left[c\left(x,y\right)\right]}^{\beta }\cdot {\left[s\left(x,y\right)\right]}^{\gamma }, \end{array}$$

where *α* > 0, *β* > 0, *γ* > 0 are parameters to adjust the relative importance of the three components. The detailed description is given in [23].

## 6. Experimental Results

**Figure 6:**
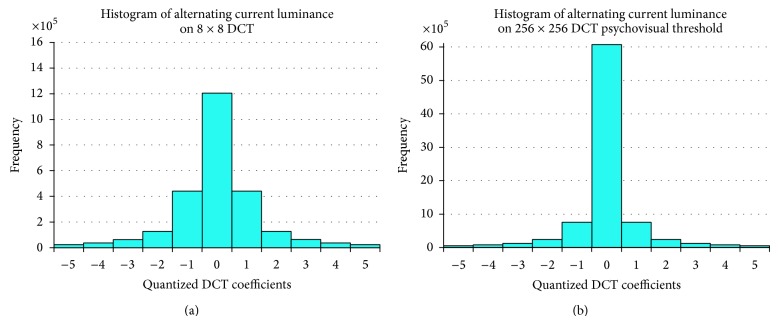
Frequency distribution of the alternating current (AC) coefficients using 8 × 8 DCT quantization (a) and 256 × 256 DCT quantization from psychovisual threshold (b) on luminance for 40 real images.

**Figure 7:**
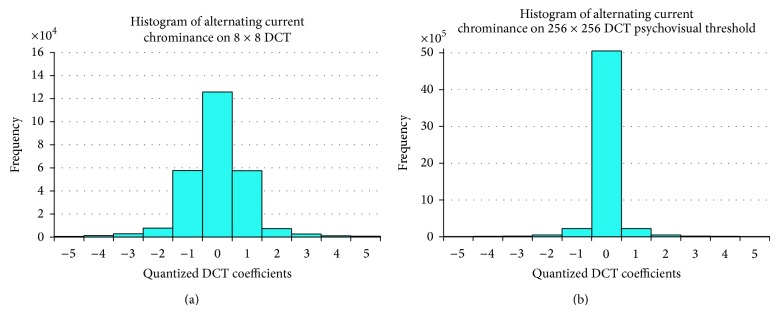
Frequency distribution of the alternating current (AC) coefficients using 8 × 8 default JPEG quantization tables (a) and 256 × 256 DCT quantization from psychovisual threshold (b) on chrominance for 40 real images.

This quantitative experiment has been conducted to investigate the performance of a psychovisual threshold on the large image block. The new finer 256 × 256 quantization tables have been generated from the psychovisual threshold on 256 × 256 DCT. They are tested on 40 real and 40 graphical high fidelity images. An input image consists of 512 × 512 colour pixels. The RGB image components are converted to YCbCr color space. In this experiment, each image is divided into 256 × 256 image block pixels; thus each block is transformed into 256 × 256 DCT. The DCT coefficients are quantized by new finer 256 × 256 quantization tables from psychovisual threshold. The frequency distribution of the transformed coefficients after the quantization process is summarised by histograms in Figures 6 and 7. The histogram of the frequency distribution is obtained after quantization processes. Figures 6 and 7 show a histogram of the frequency distribution after quantization process of 8 × 8 default JPEG quantization tables and 256 × 256 quantization tables from psychovisual threshold for luminance and chrominance, respectively.

The compression rate focuses mainly on the contribution of the AC coefficients to image compression performance. The frequency coefficients after quantization process consist of many zeros. The frequency distribution of the AC coefficients given by its histogram may predict the compression rate. The higher zeros value on the histogram of frequency distribution means that the image compression output provides lower bit rate to present an image.

According to Figures 6 and 7, the distribution of the frequency coefficients after the quantization process by smoother 256 × 256 quantization tables from psychovisual thresholds produces significantly more zeros for both luminance and chrominance channels, respectively. These finer quantization tables produce a smaller standard deviation on AC coefficients from the large 256 × 256 image block than the small 8 × 8 image block. Hence, it is possible to code large transformed block using smaller number of bits for the same image.

The 256 × 256 transform coding consists of 65535 AC coefficients for each regular block. Most AC coefficients are naturally small coming out of quantization process. The psychovisual threshold on large DCT determines an optimal contribution of the AC coefficients of large transform coding. The new 256 × 256 quantization tables from psychovisual threshold are able to reduce down the irrelevant AC coefficients. The distribution of the transform coefficients gives an indication on how much transform coefficients will be encoded by a lossless Huffman coding.

**Figure 8:**
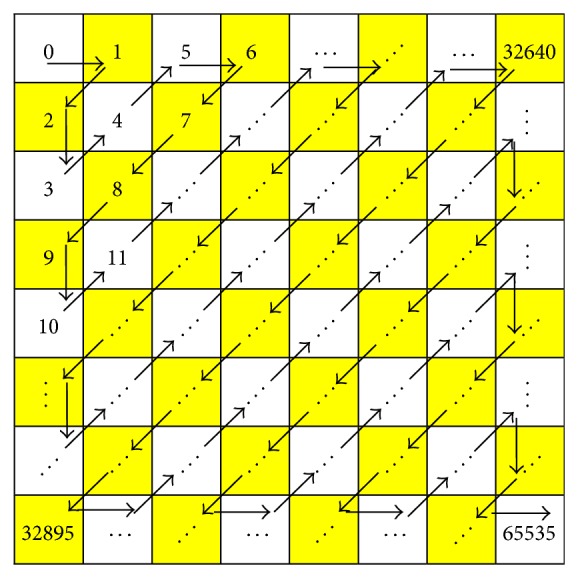
Zigzag order of 256 × 256 image block.

An average Huffman code is calculated from the quantized transform coefficients. After the transformation and quantization of 256 × 256 image block are over, the direct current (DC) coefficient is separated from the AC coefficients. The AC coefficients are listed as a traversing array in zigzag pattern as shown in Figure 8.

Next, run length encoding is used to reduce the size of a repeating coefficient in the sequence of the AC coefficients. The coefficient values can be represented compactly by simply indicating the coefficient value and the length of its run wherever it appears. The output of run length coding represents the symbols and the length of occurrence of the symbols. The symbols and variable length of occurrence are used in Huffman coding to retrieve code words and their length of code words. Using these probability values, a set of Huffman code of the symbols can be generated by Huffman tree. Next, the average bit length is calculated to find the average bit length of the AC coefficients.

There are only four DC coefficients under regular 256 × 256 DCT. The maximum code length of the DC coefficient is 16 bits. The DC coefficients are reduced down by 4 bits. The average bit length of the DC coefficients produces 12 bits after the quantization process in image compression. The average bit length of image compression based on default 8 × 8 JPEG quantization tables and the finer 256 × 256 quantization tables from psychovisual threshold is shown in Table 4. The experimental results show the new large quantization tables from psychovisual threshold produce a lower average bit length of Huffman code than the default JPEG quantization tables.

The DCT coefficients from a large image block have been greatly discounted by quantization tables in image compression. An optimal amount of DCT coefficients is investigated by reconstruction error and average bit length of Huffman code. The effect of incrementing DCT coefficient has been explored from this experiment. The average reconstruction error from incrementing DCT coefficients is mainly concentrated in the low frequency order of the image signals.

The new 256 × 256 quantization table from the psychovisual threshold produces a lower average bit length of Huffman code in image compression as shown in Table 4. At the same time, the compressed output images produce a better quality image reconstruction than the regular 8 × 8 default JPEG quantization tables as listed in Table 5. The new design on quantization tables from psychovisual threshold performs better by producing higher quality in image reconstruction at lower average bit length of Huffman code.

**Figure 9:**
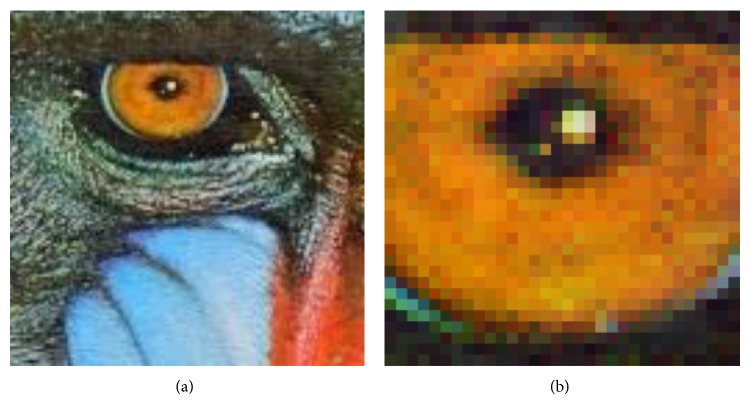
Original baboon image (a) zoomed in to 400% (b).

The average bit size of image compression as presented in Table 6 shows that the finer 256 × 256 quantization tables from psychovisual threshold use fewer bits. Therefore, the compression ratio of the difference between a compressed image from the new large quantization table and the original image produces a higher compression ratio than standard JPEG image compression as shown in Table 7. In order to observe the visual quality of the output image, a sample of original baboon right eye is zoomed in to 400% as depicted on the right of Figure 9.

**Figure 10:**
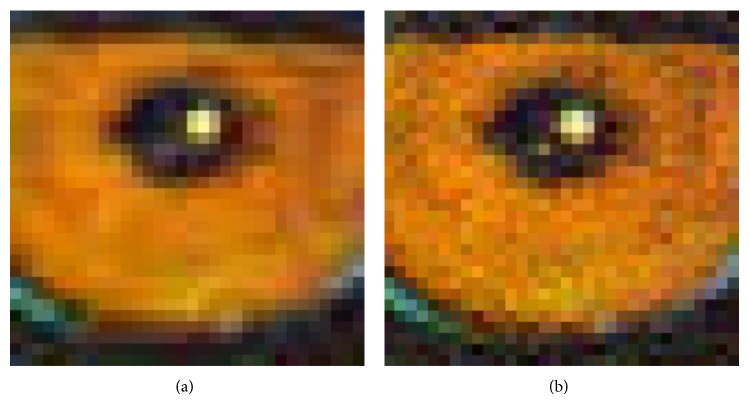
The comparison of visual outputs between 8 × 8 JPEG quantization table (a) and 256 × 256 quantization tables from psychovisual threshold (b) zoomed in to 400%.

The image compression output of 256 × 256 quantization table from psychovisual threshold is shown on the right of Figure 10. A visual inspection on the output image using 256 × 256 quantization tables from psychovisual threshold produces the richer texture on the baboon image. The psychovisual threshold on 256 × 256 image block gives an optimal balance between the fidelity on image reconstruction and compression rate. The experimental results show that the psychovisual threshold on large DCT provides minimum image reconstruction error at lower bit rates. The JPEG image compression output as depicted on the left of Figure 10 contains artifact image or blocking effect under regular 8 × 8 block transform coding. At the same time, the smoother 256 × 256 quantization tables from psychovisual threshold manage to overcome the blocking effects along the boundary blocks. These finer 256 × 256 quantization tables from psychovisual threshold provide high quality image with fewer artifact images. The psychovisual threshold is practically the best measure of an optimal amount of transform coefficients to the image coding.

## 7. Conclusion

This research project has been designed to support large block size in a practical image compression operation in the near future. A step-by-step procedure has been developed to produce psychovisual threshold on 256 × 256 block transform. The use of the psychovisual threshold on a large image block is able to overcome the blocking effect or artifact image which often occurs in standard JPEG compression. The psychovisual threshold on discrete transform has been used to determine an optimal amount of frequency transform coefficients to code by generating the much needed quantization tables. Naturally, a frequency transform on large image block is capable of reducing redundancy and better exploiting pixel correlation within the image block. The new set of quantization tables from psychovisual threshold produces better performance than JPEG image compression. These quantization tables from psychovisual threshold practically provide higher quality images at lower bit rate for image compression application.

## Figures and Tables

**Table 1:** The index of quantization value on each frequency order.

0	1	5	6	14	15	27	28	44	45	65	66	90	91	119	120	152	153	189	190	230	231	275
2	4	7	13	16	26	29	43	46	64	67	89	92	118	121	151	154	188	191	229	232	274	276
3	8	12	17	25	30	42	47	63	68	88	93	117	122	150	155	187	192	228	233	273	277	318
9	11	18	24	31	41	48	62	69	87	94	116	123	149	156	186	193	227	234	272	278	317	319
10	19	23	32	40	49	61	70	86	95	115	124	148	157	185	194	226	235	271	279	316	320	357
20	22	33	39	50	60	71	85	96	114	125	147	158	184	195	225	236	270	280	315	321	356	358
21	34	38	51	59	72	84	97	113	126	146	159	183	196	224	237	269	281	314	322	355	359	392
35	37	52	58	73	83	98	112	127	145	160	182	197	223	238	268	282	313	323	354	360	391	393
36	53	57	74	82	99	111	128	144	161	181	198	222	239	267	283	312	324	353	361	390	394	423
54	56	75	81	100	110	129	143	162	180	199	221	240	266	284	311	325	352	362	389	395	422	424
55	76	80	101	109	130	142	163	179	200	220	241	265	285	310	326	351	363	388	396	421	425	450
77	79	102	108	131	141	164	178	201	219	242	264	286	309	327	350	364	387	397	420	426	449	451
78	103	107	132	140	165	177	202	218	243	263	287	308	328	349	365	386	398	419	427	448	452	473
104	106	133	139	166	176	203	217	244	262	288	307	329	348	366	385	399	418	428	447	453	472	474
105	134	138	167	175	204	216	245	261	289	306	330	347	367	384	400	417	429	446	454	471	475	492
135	137	168	174	205	215	246	260	290	305	331	346	368	383	401	416	430	445	455	470	476	491	493
136	169	173	206	214	247	259	291	304	332	345	369	382	402	415	431	444	456	469	477	490	494	507
170	172	207	213	248	258	292	303	333	344	370	381	403	414	432	443	457	468	478	489	495	506	508
171	208	212	249	257	293	302	334	343	371	380	404	413	433	442	458	467	479	488	496	505	509
209	211	250	256	294	301	335	342	372	379	405	412	434	441	459	466	480	487	497	504	510
210	251	255	295	300	336	341	373	378	406	411	435	440	460	465	481	486	498	503	
252	254	296	299	337	340	374	377	407	410	436	439	461	464	482	485	499	502	
253	297	298	338	339	375	376	408	409	437	438	462	463	483	484	500	501	

**Table 2:** The quantization value on each frequency order for luminance.

16	12	9	9	7	7	7	7	7	7	7	7	8	8	9	9	11	11	16	16	23	23	34
11	9	8	8	7	7	7	7	7	7	7	8	8	9	9	11	11	16	16	23	23	34	34
10	8	8	7	7	7	7	7	7	7	8	8	9	9	11	11	15	16	23	24	34	35	47
8	8	7	7	7	7	7	7	7	8	8	9	9	11	12	15	16	22	24	33	35	46	47
8	7	7	7	7	7	7	7	7	8	8	9	11	12	15	16	22	24	33	35	46	47	58
7	7	7	7	7	7	7	7	8	8	9	11	12	15	17	22	24	33	35	46	48	58	58
7	7	7	7	7	7	7	8	8	9	11	12	15	17	22	25	33	36	45	48	58	59	66
7	7	7	7	7	7	8	8	9	11	12	15	17	22	25	32	36	45	48	57	59	66	66
7	7	7	7	7	8	8	9	10	12	15	17	21	25	32	36	45	48	57	59	66	66	68
7	7	7	7	8	8	9	10	12	15	17	21	25	32	37	45	49	57	59	65	66	68	68
7	7	7	8	8	9	10	12	14	17	21	26	32	37	44	49	57	60	65	66	68	69	65
7	7	8	8	9	10	13	14	18	21	26	32	37	44	49	56	60	65	67	68	69	66	65
7	8	8	10	10	13	14	18	21	26	31	37	44	50	56	60	65	67	68	69	66	65	56
8	8	10	10	13	14	18	20	26	31	38	43	50	56	60	65	67	68	68	66	64	57	56
8	10	10	13	14	18	20	27	31	38	43	50	56	61	64	67	68	68	66	64	57	55	42
10	10	13	14	18	20	27	31	38	43	51	56	61	64	67	68	68	66	64	58	55	43	41
10	13	14	18	20	27	31	39	42	51	55	61	64	67	68	68	67	64	58	54	44	40	27
13	14	19	20	28	31	39	42	51	55	61	64	67	68	68	67	63	59	53	45	39	28	26
13	19	20	28	30	39	42	52	55	62	64	68	68	68	67	63	59	53	46	38	29	24
19	19	29	30	39	41	52	55	62	63	68	68	68	67	63	60	52	47	37	30	20
19	29	30	40	41	53	55	62	63	68	68	68	67	62	60	51	48	36	31	
29	30	40	41	53	54	62	63	68	68	68	68	62	61	51	48	35	32	
30	40	41	54	54	62	63	68	68	68	68	61	61	50	49	34	33	

**Table 3:** The quantization value on each frequency order for chrominance.

17	13	10	9	9	8	8	8	8	8	9	9	10	10	12	12	17	18	27	27	43	44	68
12	10	9	9	8	8	8	8	8	9	9	10	10	12	12	17	18	27	28	43	44	67	69
11	9	9	8	8	8	8	8	9	9	9	10	12	12	17	18	26	28	42	45	67	69	97
9	9	8	8	8	8	8	9	9	9	10	12	12	17	18	26	28	42	45	66	70	96	97
9	8	8	8	8	8	9	9	9	10	12	13	16	18	26	29	41	46	66	70	95	98	123
8	8	8	8	8	9	9	9	10	11	13	16	19	26	29	41	46	65	71	94	99	123	124
8	8	8	8	9	9	9	10	11	13	16	19	25	29	40	47	65	72	94	99	122	125	142
8	8	9	9	9	9	10	11	13	16	19	25	30	40	47	64	72	93	100	122	125	142	143
8	9	9	9	9	10	11	13	16	19	25	30	40	48	64	73	92	101	121	126	142	143	149
9	9	9	9	10	11	13	16	20	24	30	39	48	63	73	92	102	121	126	141	143	149	150
9	9	9	10	11	13	15	20	24	31	39	49	63	74	91	102	120	127	141	144	149	150	143
9	9	10	11	14	15	20	24	31	38	49	62	75	91	103	119	128	141	144	149	150	143	142
9	10	11	14	15	21	24	32	38	50	62	75	90	104	119	129	140	145	149	150	145	141	122
11	11	14	15	21	23	32	37	50	61	76	89	105	118	129	140	145	149	149	144	141	123	121
11	14	15	21	23	32	37	51	61	77	89	105	118	130	139	145	149	149	145	140	124	119	89
14	14	21	23	33	37	52	61	77	88	106	118	130	139	146	149	149	145	140	126	118	91	87
14	22	23	33	36	52	60	78	87	107	117	131	138	146	149	149	146	139	127	116	93	84	48
22	23	33	36	53	60	79	86	108	116	131	138	146	149	149	146	138	128	115	95	82	51	46
22	34	35	54	59	79	85	109	116	132	137	147	149	149	146	137	129	113	97	79	54	43
34	35	55	59	80	85	109	115	132	136	147	149	149	147	137	130	112	99	77	57	41
35	56	58	81	84	110	114	133	136	147	149	148	147	136	131	110	101	74	60	
56	58	81	83	111	114	134	135	148	149	148	148	135	132	108	103	72	63	
57	82	83	112	113	134	135	148	149	148	148	134	133	107	105	69	66	

**Table 4:** Average bit length of Huffman code of image compression using 8 × 8 JPEG compression and 256 × 256 JPEG compression using psychovisual threshold for 40 real images and 40 graphical images.

Average bit length of Huffman code	8 × 8 JPEG compression		256 × 256 JPEG compression	
	40 real images	40 graphic images	40 real images	40 graphic images
DC luminance	5.7468	5.6722	12	12
DC chrominance Cr	2.7941	3.8663	12	12
DC chrominance Cb	3.1548	4.0730	12	12
AC luminance	2.8680	2.9653	2.3031	2.6582
AC chrominance Cr	2.0951	2.5059	1.2931	2.1450
AC chrominance Cb	2.1845	2.5158	1.3656	2.1855

**Table 5:** The average image reconstruction error using 8 × 8 JPEG compression and 256 × 256 JPEG compression using psychovisual threshold for 40 real images and 40 graphical images.

Image measurement	8 × 8 JPEG compression		256 × 256 JPEG compression	
	40 real images	40 graphic images	40 real images	40 graphic images
ARE	5.535	5.648	5.074	5.050
MSE	70.964	92.711	51.841	59.625
PSNR	31.190	31.636	32.363	33.421
SSIM	0.956	0.957	0.961	0.960

**Table 6:** The average bit size of 8 × 8 JPEG compression and 256 × 256 JPEG compression using psychovisual threshold.

8 × 8 JPEG compression		256 × 256 JPEG compression	
40 real images	40 graphic images	40 real images	40 graphic images
230.997 Kb	258.394 Kb	158.792 Kb	223.652 Kb

**Table 7:** The average compression ratio score of 8 × 8 JPEG compression and 256 × 256 JPEG compression using psychovisual threshold.

8 × 8 JPEG compression		256 × 256 JPEG compression	
40 real images	40 graphic images	40 real images	40 graphic images
3.3247	2.9722	4.8365	3.4339
